# Identification of a signature gene set for oxaliplatin sensitivity prediction in colorectal cancer

**DOI:** 10.3389/fonc.2025.1701328

**Published:** 2025-11-27

**Authors:** Xiaopeng Zhan, Xinyue Li, Yimin Chu, Ying Xu, Fengli Zhou, Ji Li, Daming Yang, Changping Hu, Haixia Peng, Zhaoxia Wu

**Affiliations:** 1 Digestive Endoscopy Center, Shanghai Tongren Hospital, Shanghai Jiaotong University School of Medicine, Shanghai, China; 2 Digestive Endoscopy Center, Key Laboratory for Translational Research and Innovative Therapeutics of Gastrointestinal Oncology, Hongqiao International Institute of Medicine, Tongren Hospital, Shanghai Jiao Tong University School of Medicine, Shanghai, China

**Keywords:** colorectal cancer, oxaliplatin sensitivity, machine learning algorithms, gene set, prognostic biomarkers

## Abstract

**Background:**

Colorectal cancer (CRC) is a leading cause of cancer-related mortality worldwide. Oxaliplatin-based chemotherapy is a cornerstone of treatment for many CRC patients; however, the development of chemoresistance severely limits its therapeutic efficacy and remains a major clinical challenge. The identification of robust biomarkers to predict oxaliplatin sensitivity is therefore critical for personalizing treatment and improving patient outcomes.

**Methods:**

Here, we conducted a comprehensive analysis of large-scale genomic datasets, including from the Gene Expression Omnibus (GEO) and The Cancer Genome Atlas (TCGA). Machine learning algorithms to these datasets was applied to identify genes associated with oxaliplatin response. The prognostic value of the candidate genes was evaluated using progression-free survival (PFS) analysis. The predictive robustness of the identified gene set was further validated in external datasets from GEO and the Genomics of Drug Sensitivity in Cancer (GDSC) database using multivariate logistic regression analysis. Finally, we experimentally assessed the functional role of these genes by examining their expression in oxaliplatin-resistant cell lines and by performing gene knockdown experiments in colorectal cancer cells to measure subsequent changes in oxaliplatin sensitivity.

**Results:**

Our integrated bioinformatics approach identified 14 genes potentially linked to oxaliplatin sensitivity. Subsequent PFS analysis narrowed this set to four key genes (AXDND1, BAMBI, MAPK8IP2, and BMP7) that were significantly associated with patient survival following oxaliplatin-based therapy. External validation confirmed that different combinations of these four genes consistently and robustly predicted oxaliplatin sensitivity. Furthermore, the expression levels of these four genes were significantly altered in both independent validation datasets and in established oxaliplatin-resistant cell lines. Functional studies demonstrated that silencing these genes directly influenced oxaliplatin cytotoxicity: knockdown of MAPK8IP2 significantly enhanced oxaliplatin-induced cell death, whereas knockdown of BAMBI and BMP7 significantly reduced cellular sensitivity to the drug.

**Conclusion:**

Our study identifies a novel four-gene signature that is strongly associated with oxaliplatin sensitivity in colorectal cancer. These findings support developing prognostic biomarkers to optimize oxaliplatin use, aiming to identify sensitive patients and avoid treatment for those with resistance.

## Introduction

Colorectal cancer (CRC) is a significant global health problem. It is the third most commonly diagnosed cancer and the second leading cause of cancer-related deaths worldwide, with approximately 2.0 million new cases and 0.9 million deaths globally (1). This underscores the critical need for effective treatment modalities and the continuous evolution of oncologic therapies. The deployment of oxaliplatin-based chemotherapy has been a significant advancement in CRC management, offering hope to patients with advanced disease stages (2, 3). Despite its initial efficacy, the clinical utility of oxaliplatin is hampered by the development of chemoresistance (4). The ability of cancer cells to evade the cytotoxic effects of oxaliplatin results in a reduction in therapeutic efficacy and a subsequent unfavorable prognosis.

Recent research has elucidated the multifaceted molecular mechanisms that underpin oxaliplatin resistance in CRC, underscoring the intricate nature of this phenomenon. It is imperative to consider the following factors: alterations in drug transport and detoxification processes, with particular reference to the cellular uptake and efflux of oxaliplatin. These factors have been shown to markedly influence drug availability and treatment efficacy (5). Furthermore, enhanced DNA repair mechanisms serve to reinforce resistance, particularly through the activation of the Ataxia-Telangiectasia Mutated and RAD3-Related (ATR) protein kinase. Inhibition of this process has been demonstrated to restore drug sensitivity (6). Additionally, epigenetic changes such as miRNA, lncRNA regulation and methylation significantly modify gene expressions linked to resistance (7–10). The tumor microenvironment also plays a crucial role, with components like myeloid-derived suppressor cells and macrophages potentially being manipulated to alter the tumor’s chemotherapy response (11, 12). Moreover, resistance can emerge from modifications in cell death pathways, including apoptosis and necroptosis. Molecular triggers have been identified that could serve as potential targets to counteract resistance (13). It is evident that the resistance to oxaliplatin chemotherapy is multifactorial, encompassing intricate genetic, epigenetic, and environmental interactions that diminish drug efficacy. The current approaches to counteract this resistance are constrained by a limited understanding of these complex mechanisms.

The objective of this study was to identify the molecular determinants of oxaliplatin sensitivity in CRC. The employment of extensive transcriptomic data and advanced computational methodologies, followed by the validation of external datasets, has enabled the identification of four genes (*AXDND1, BAMBI, MAPK8IP2 and BMP7*) that are associated with PFS following oxaliplatin-based therapy. The utilization of these genes in different combinations has been demonstrated to serve as potential predictive markers for oxaliplatin sensitivity. The results of gene-silencing experiments demonstrated that the four genes modulate the sensitivity of colorectal cancer cells to oxaliplatin. These findings not only expand our recognition of the genetic signatures of oxaliplatin resistance in CRC, but also provide clues for future precision treatment of CRC.

## Materials and methods

### Data acquisition and processing

We systematically searched the Gene Expression Omnibus (GEO) database using the following keywords: ‘colorectal cancer’ and ‘oxaliplatin resistance’, The inclusion criteria for datasets were: (1) specifically focus on oxaliplatin resistance in CRC, containing gene expression profiles of oxaliplatin-resistant Human CRC cell lines or CRC patients samples; (2) offer complete profiles with corresponding clinical or drug sensitivity metadata; (3)including matched sensitive controls (e.g., parental cell lines or treatment-sensitive patients); (4) sample size ≥ 3 per group. (5) Public before November 1, 2023. The following datasets were selected: GSE119603 (HCT116_oxR vs. HCT116), GSE76092 (HT29_oxR vs. HT29) (14) and GSE42387 (LOVO_oxR vs. LOVO) (15) datasets, GSE83129 (16) and GSE28702 (17) (oxaliplatin-treated CRC patients). All data were downloaded using the “GEOquery” R package (version 2.72.0). For TCGA, we retrieved RNA-seq data and clinical information of 106 oxaliplatin-treated CRC patients from the TCGA-COADREAD project using the “TCGAbiolinks” R package (version 2.31.1). Only samples with complete clinical annotation and treatment records were included.

The data of tumor cell lines treated with oxaliplatin was obtained from the Genomics of Drug Sensitivity in Cancer (GDSC) database (www.sanger.ac.uk/), which includes the name of each cell line, their IC50 values, and the transcript expression profiles after oxaliplatin treatment. Additionally, the RNA-seq dataset containing 60 diverse human tumor cell lines and corresponding oxaliplatin drug sensitivity information was downloaded from the CellMiner database (https://discover.nci.nih.gov/cellminer/).

### Identifying differentially expressed genes

The DESeq2 package (version 1.40.0) was employed to detect differentially expressed genes (DEGs) between 19 oxaliplatin-resistant and 28 oxaliplatin-sensitive CRC patients in the TCGA-COADREAD dataset. Furthermore, DESeq2 was utilized to identify DEGs between 3 oxaliplatin-resistant CRC cells (HCT116_oxR) and 3 control HCT116 cells in the GSE119603 dataset. |Log2 fold-change| ≥ 1 and P-value < 0.05 were set as the selection criteria to define the scope of DEGs in two databases for further analysis. Additionally, volcano plots were drawn using the R packages “limma” (version 3.60.4) and “ggplot2” (version 3.5.1). The gene annotation and analysis resource website “Metascape” (https://www.metascape.org/) was utilized to cluster and enrich pathways for the intersecting DEGs(co-DEGs) of two databases.

### Pathway enrichment analyses

To comprehensively evaluate biological pathways associated with oxaliplatin resistance while minimizing analytical bias, we performed integrated enrichment analyses on both the 48 common DEGs and the full DEG sets from TCGA-COADREAD and GSE119603. Gene Set Enrichment Analysis (GSEA v4.1.0) was conducted on ranked gene lists (by log2FC) using the MSigDB Hallmark gene set (h.all.v2025.1.Hs.symbols.gmt), with significance defined as FDR q-value < 0.25 and |NES| > 1.0; all genes with P-value < 0.05 were included to enhance pathway-level sensitivity. Parallel KEGG pathway analysis was performed using the “clusterProfiler”(version 4.10.0) R package, considering pathways with Benjamini-Hochberg adjusted p-value < 0.05 as significant. Additionally, the 48 common DEGs underwent functional annotation through Metascape with default parameters (significance thresholds: p < 0.01, minimum count = 3, enrichment factor > 1.5).

### Screening and validation of oxaliplatin resistance-related genes based on co-DEGs

Novel and significant biomarkers for oxaliplatin resistance in CRC patients who underwent oxaliplatin based chemotherapy in TCGA-COADREAD program were screened by integrating the co-DEGs into three machine learning algorithms: Least Absolute Shrinkage and Selection Operator (LASSO), Random Forest (RF), and Support Vector Machine (SVM). The R package ‘glmnet’ (version 4.1.8) was used for the LASSO algorithm, The R package ‘e1071’ (version 1.7.14) was applied to the SVM algorithm with a ‘linear’ kernel. The R package ‘randomForest’ (version 4.7.1.1) was adopted for the RF algorithm. All models employed 5-fold cross-validation to optimize hyperparameters and prevent overfitting. Subsequently, genes identified from the three machine learning algorithms were overlapped to obtain common genes.

Kaplan–Meier curves were generated to visualize the progression-free survival (PFS) of patients receiving oxaliplatin-based therapy in the TCGA-COADREAD cohort stratified by high and low expression of the common genes. The statistical significance of the differences in the survival curves was assessed using a log-rank test, and a p-value < 0.05 was regarded statistically significant. The genes associated with PFS were identified as key genes and then selected for additional study.

The expression profiles of the key genes were initially verified using qRT-PCR and external datasets (GSE76092 and GSE42387), which encompassed data from two CRC oxaliplatin-resistant cell lines along with their respective control cohorts.

To thoroughly evaluate the utility of key genes in predicting oxaliplatin sensitivity, this study selected datasets GSE83129, GSE28702, and a subset of the GDSC as validation sets. Logistic regression models were built by R package ‘glmnet’ and different gene combination models and their Receiver Operating Characteristic-Area Under the Curve (ROC-AUC) values were listed by R package “mclust”. The best gene combinations and their AUC values of the three validation sets were displayed with Receiver Operating Characteristic (ROC) curves respectively. Statistical significance was determined using a two-tailed test, with a P-value below 0.05 considered statistically significant. Discrimination by the ROC-AUC values is commonly assessed as: poor (<0.60), moderate (0.60-0.75), or good to excellent (>0.75) (18).

### Cell culture, transfections, lentiviral infection

The human embryonic kidney (HEK) 293T cell lines were purchased from the American Type Culture Collection and maintained in Dulbecco’s modified Eagle’s medium supplemented with 10% fetal bovine serum. HT29 and SW1116 cells were obtained from Shanghai Zhongqiao Xinzhou Biotechnology Co. HCT116 and oxaliplatin-resistant CRC cells (HCT116_oxR) were obtained from Shanghai Meixuan Biotechnology Co. HT29 cells were cultured in McCoy’s basic medium supplemented with 10% fetal bovine serum. SW1116, HCT116 and HCT116_oxR cells were maintained in RPMI 1640 medium supplemented with 10% fetal bovine serum. Lentiviral supernatants for short hairpin RNA (shRNA) were produced by co-transfecting 293T cells with the construct pLKO.1-puro-vector and packing plasmids pMD2.G and psPAX2. When the cells were growing to 40-60% confluence, HT29 and SW1116 cells were infected with the viral supernatants. Following a 48-hour incubation period, 2 μg/ml puromycin was added to the medium, and the stable cells were selected. RT-qPCR was subsequently performed to evaluate targeted gene expression and validate knockdown efficiency. Information on the shRNAs is shown in **Supplementary Table S1**.

### Real-time quantitative reverse transcription PCR

Total mRNA was isolated using the FastPure Cell/Tissue Total RNA Isolation Kit V2. The concentration, quality, and integrity were then determined using a NanoDrop spectrophotometer (Thermo Scientific). cDNA was then synthesized from the extracted total RNA using the HiScript III RT SuperMix for qPCR (+gDNA wiper) Kit. Quantitative PCR (Q-PCR) was conducted using the ChamQ Universal SYBR qPCR Master Mix Kit. The relative mRNA expression levels were determined using the 2^−ΔΔCT method. These experiments were replicated independently three times. Both kits used were obtained from Vazyme(China), Glyceraldehyde 3-phosphate dehydrogenase (GAPDH) was utilized as an internal reference gene for normalizing the data. Primers used for quantitative real-time PCR are listed in **Supplementary Table S2**.

### Oxaliplatin intervention and cell viability assay

The gene knockdown cell lines along with their respective negative controls were maintained in RPMI1640 medium enriched with 10% fetal bovine serum (FBS) and an antibiotic mix of 100 μg/ml penicillin-streptomycin. Once the cells reached approximately 80% confluence, they were harvested from both cell lines, re-suspended, and counted. Subsequently, the cells were seeded into 96-well plates at a density of 8×10^3 cells per well. Oxaliplatin exposure followed, with escalating concentrations for HT29 cells set at 0 μg/ml, 5 μg/ml, 10 μg/ml, and 20 μg/ml, whereas for SW1116 cells, the concentrations were 0 μg/ml, 10 μg/ml, 20 μg/ml, and 40 μg/ml. The duration of exposure for both cell types was 48 hours. Post-treatment, the cells were incubated for an additional 2 hours in complete culture medium containing 10% CCK8 solution. Cell viability was then determined by measuring the optical density (OD) at 450 nm using a TECAN Infinite 200 PRO microplate reader.

### Statistical analysis

This research was primarily conducted utilizing R (Version 4.3.2) and RStudio software. We employed a variety of R packages, including “GEOquery”, “DESeq2”, “readr”, “dplyr”, “rtracklayer”, “limma”, “pacman”, “TCGAbiolinks”, “scRNAseq”, “data.table”, “broom”, “glmnet”, “VennDiagram”, “sigFeature”, “e1071”, “caret”, “randomForest”, “shape”, “survival”, “survminer”, “mclust”, “pROC”, and “SimDesign”. The progression-free survival (PFS) between two groups was assessed using the Kaplan-Meier survival analysis and log-rank test. Mean values were compared using the two-tailed Student’s t-test in GraphPad Prism 8. Unless otherwise specified, statistical significance was defined as P <0.05. To further assess the independent prognostic value of the four-gene signature (A*XDND1, BAMBI, BMP7*, and *MAPK8IP2*), multivariable Cox proportional hazards regression was conducted using the “survival” R package (version 3.5.7). Continuous mRNA expression values (log_2_-transformed TPM) of the four genes were included in the model, alongside clinical covariates: age (≥60 vs. <60 years), sex (male = 1, female = 0), and TNM stage (early-stage I/II vs. advanced-stage III/IV), classified according to the AJCC 8th edition. The proportional hazards assumption was tested using Schoenfeld residuals via the cox.zph function, with no significant violations detected (all p > 0.05). Hazard ratios (HRs) and 95% confidence intervals (CIs) were calculated to estimate the independent association of each variable with progression-free survival (PFS), and statistical significance was defined as p < 0.05 (two-tailed).

## Results

### Screening of differentially expressed genes and pathway alterations in oxaliplatin resistance

**Figure 1:**
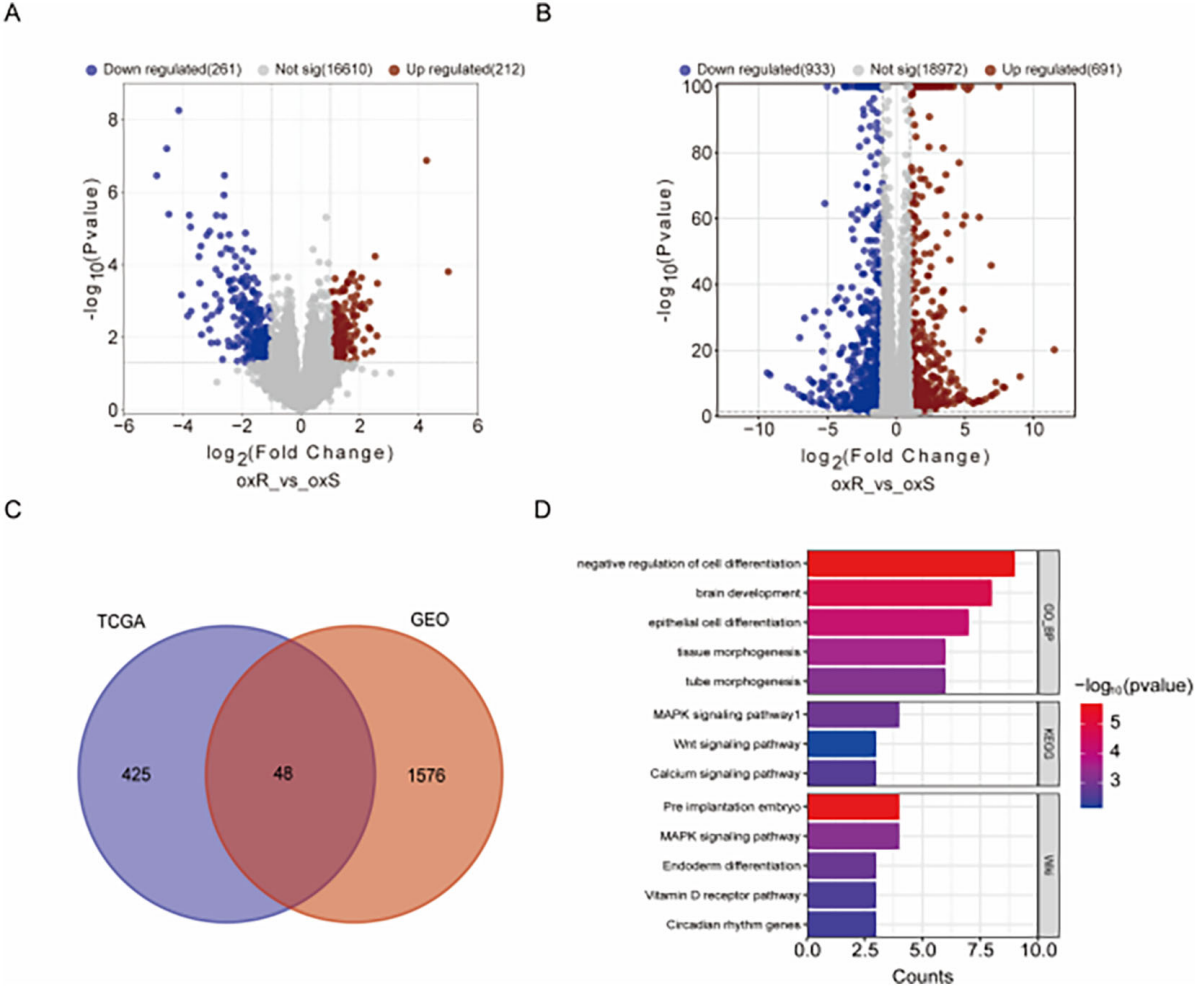
Genetic Profiling and Analysis of oxaliplatin Resistance. **(A)** Volcano plot comparison between oxaliplatin-resistant and control tumor tissues in the TCGA-CO/READ database, using |Log2 fold-change| ≥ 1 and P-value < 0.05 as thresholds. Red dots signify upregulated genes; blue dots indicate downregulated genes. **(B)** Volcano plot for oxaliplatin-resistant and control cells (GSE119603), employing the same thresholds and color codes. **(C)** Venn diagram highlighting the overlap of differentially expressed genes between TCGA and GEO databases, identifying 48 commonly differentially expressed genes. **(D)** Analysis of the commonly differentially expressed genes through Metascape for pathway enrichment.

Analysis of 47 oxaliplatin-treated CRC patients from TCGA (19 resistant, 28 sensitive) identified 212 upregulated and 261 downregulated genes in the resistant group, using thresholds of |Log2 fold-change| ≥ 1 and P-value < 0.05. The volcano plot (**Figure 1A**) visually summarizes this distribution, where the red and blue dots, representing upregulated and downregulated genes respectively, are clearly separated from the mass of non-significant grey dots. Similarly, comparing oxaliplatin-resistant HCT116_oxR cells to parental HCT116 (GSE119603) revealed 691 upregulated and 933 downregulated genes (**Figure 1B**). Cross-comparison yielded 48 common differentially expressed genes (DEGs) (**Figure 1C**). Functional enrichment analysis (Metascape) of these co-DEGs highlighted significant associations with cell differentiation and the MAPK signaling pathway (**Figure 1D**). Gene Set Enrichment Analysis (GSEA) of the full DEG lists from both TCGA patients (**Supplementary Figures S1A–E**) and HCT116_oxR cells (**Supplementary Figures S1F–I**) consistently revealed significant enrichment (NES>1, FDR q<0.25) for pathways including Epithelial-Mesenchymal Transition (EMT), TNFα/NFκB signaling, and UV response suppression. Complementary KEGG pathway analysis (adj. p<0.05) further confirmed enrichment in Wnt, MAPK, and Ras signaling pathways (**Supplementary Figure S2**), functionally interconnected with EMT, cell survival, and proliferation.

### Machine learning selection of gene signatures

To identify the most predictive features from the 48 co-DEGs for classifying oxaliplatin resistance, we employed three distinct machine learning algorithms on the TCGA patient dataset (19 resistant vs. 28 sensitive cases), each providing a unique approach to feature selection.

**Figure 2:**
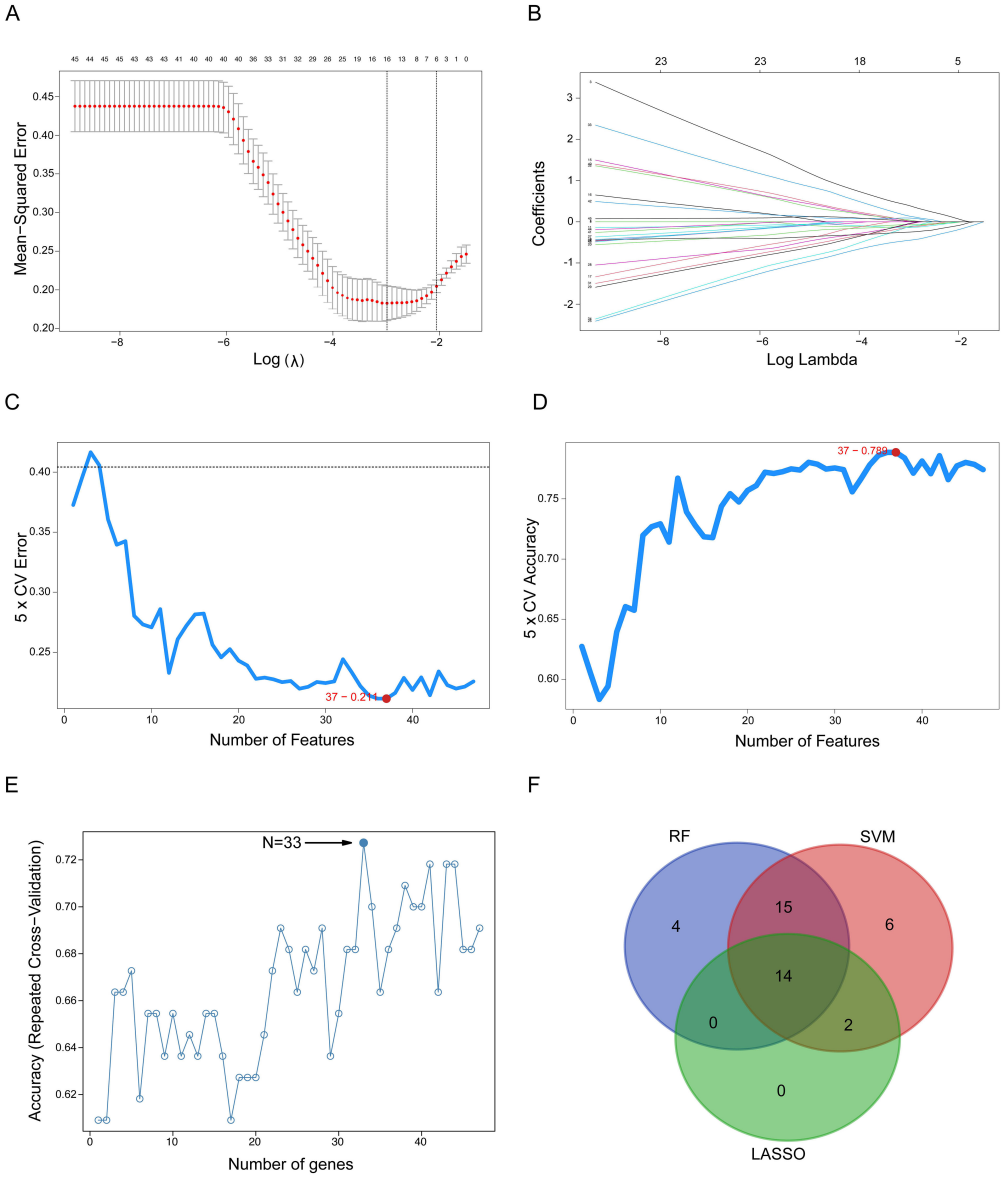
Detection of oxaliplatin resistance-related gene signatures using machine learning techniques. **(A) **The LASSO logistic regression algorithm identifies gene signatures associated with oxaliplatin resistance. **(B)** Different colors indicate distinct genes. **(C, D)** Screening of oxaliplatin resistance-related gene features using SVM. **(E)** RF-based algorithm employed to detect oxaliplatin resistance-related gene signatures. **(F)** A Venn diagram illustrates the overlap of gene signatures identified by the LASSO, SVM, and RF algorithms. SVM, Support Vector Machine; RF, Random Forest; LASSO, Least Absolute Shrinkage and Selection Operator.

First, we applied LASSO regression, which performs feature selection by applying a penalty that shrinks the coefficients of less relevant variables toward zero. In **Figure 2A**, the vertical dashed line indicates the optimal regularization parameter (lambda.min) selected via 5-fold cross-validation, which corresponds to the most parsimonious model achieving minimal cross-validation error. At this optimal lambda, the LASSO model retained 16 genes with non-zero coefficients, identifying them as the most predictive features of drug resistance. **Figure 2B** displays the coefficient paths for all 48 genes as the penalty parameter (lambda) increases: moving from left to right along the x-axis, coefficients of genes contributing less to classification are progressively shrunk to zero.

Second, we applied SVM combined with Recursive Feature Elimination (RFE). The SVM-RFE algorithm iteratively eliminates the least important features to identify an optimal feature subset that simultaneously maximizes classification accuracy and minimizes classification error. **Figure 2C** illustrates the trend of cross-validation error as a function of the number of retained features, while **Figure 2D** shows the corresponding cross-validation accuracy. Notably, the minimum of the error curve in **Figure 2C** and the peak of the accuracy curve in **Figure 2D** both correspond to an optimal gene subset comprising 37 genes.

Third, we applied RF combined with RFE identified an optimal 33-gene signature that achieved peak classification accuracy through cross-validation. The analysis demonstrated that this 33-gene subset provided the optimal balance between model performance and feature complexity, with the accuracy curve reaching its maximum at this specific gene number (**Figure 2E**).

Finally, to derive a robust and consensus gene signature, we identified the intersection of the gene sets selected by the three independent algorithms. The Venn diagram in **Figure 2F** illustrates this overlap, revealing a core set of 14 genes that were consistently selected by LASSO, SVM, and RF. This convergence across multiple algorithms strongly suggests that these 14 genes constitute a minimal yet powerful signature for predicting oxaliplatin resistance in colorectal cancer.

### Prognostic relevance of the 14-gene signature

Having identified a 14-gene resistance signature, we evaluated its clinical relevance by analyzing PFS in 106 oxaliplatin-treated CRC patients from TCGA-COADREAD. Patients were stratified by median expression of each gene.

**Figure 3:**
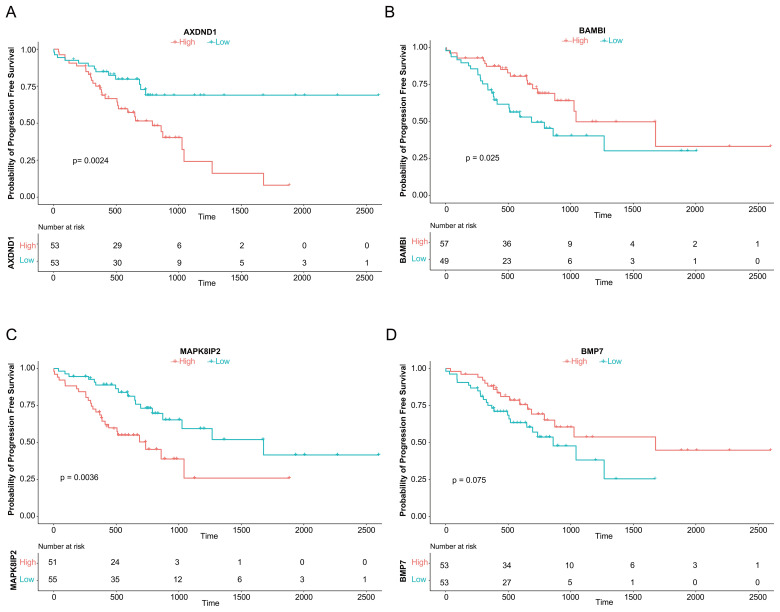
Association of Four Genes with Progression-Free Survival in CRC Patients Treated with Oxaliplatin. The analysis of progression-free survival (PFS) using median expression levels, as depicted by survival curves, demonstrated an association between the genes AXDND1 **(A)**, BAMBI **(B)**, MAPK8IP2 **(C)**, and BMP7 **(D)** and PFS in 106 TCGA patient samples that underwent oxaliplatin chemotherapy.

Kaplan-Meier analysis revealed that high expression of *AXDND1* (p = 0.024, **Figure 3A**) and *MAPK8IP2* (p = 0.0036, **Figure 3C**) was significantly associated with shorter PFS. While high *BAMBI* (p = 0.025, **Figure 3B**) and *BMP7* (p = 0.075, **Figure 3D**) expression showed a trend toward better survival. The remaining ten genes showed no significant association with PFS.

Based on these findings, we prioritized *AXDND1, BAMBI, MAPK8IP2*, and *BMP7* for further validation due to their clear prognostic relevance.

### Evaluating of predictive efficacy of key genes

**Figure 4:**
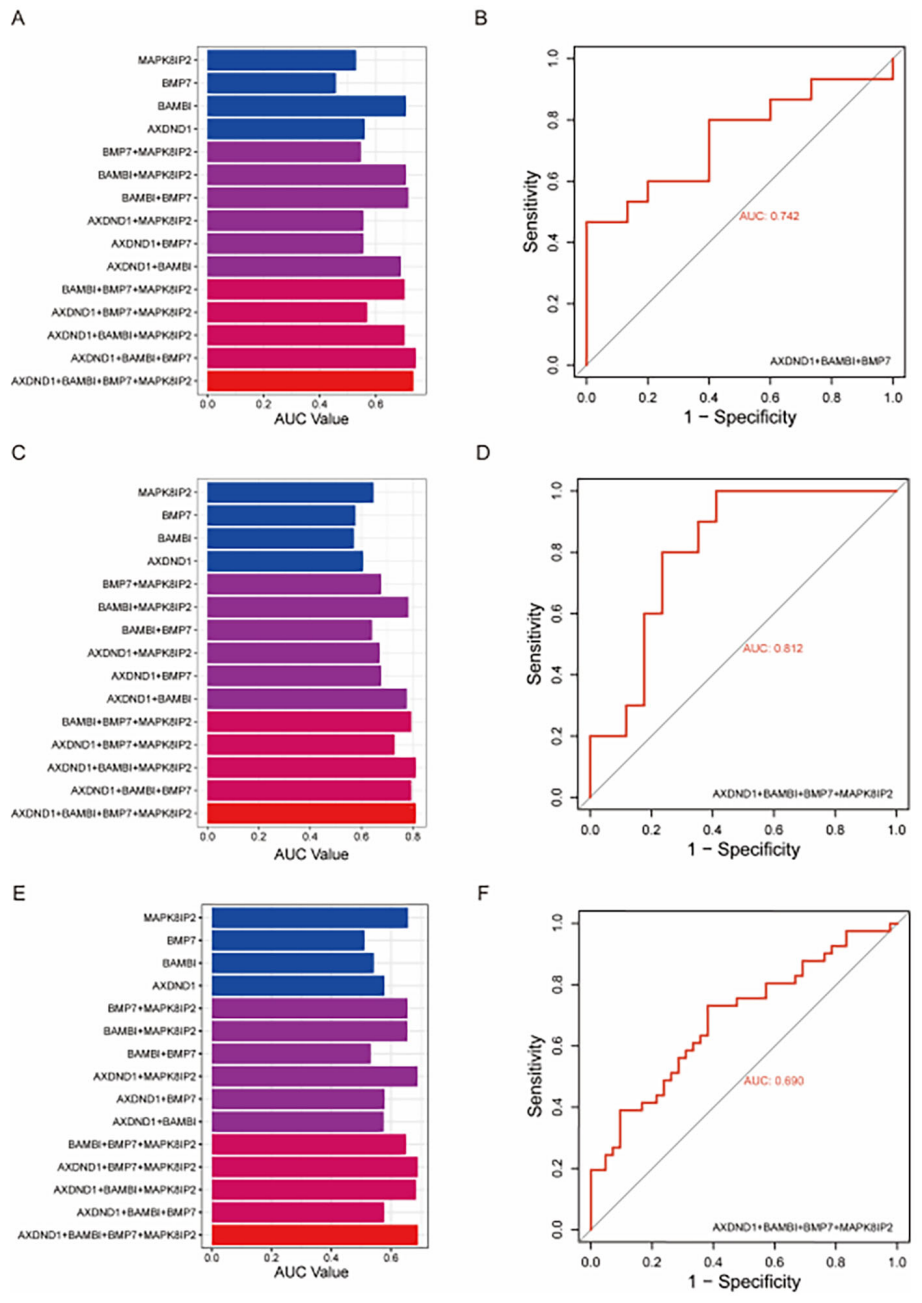
Validating the diagnostic value of oxaliplatin-resistance gene signature in other datasets of colorectal tumors and colorectal cell lines. To validate the effectiveness of the four oxaliplatin-resistant genes, we utilized external datasets and assessed their diagnostic capability for oxaliplatin resistance through multivariate logistic regression. In the GDSC dataset, the combination of AXDND1, BAMBI, and BMP7 achieved the maximum area under the curve (AUC) **(A)**, with an AUC value of 0.742 **(B)**. In the GSE83129 dataset, the combination of the four genes reached the maximum AUC **(C)**, with an AUC value of 0.812 **(D)**. In the GSE28702 dataset, the combination of the four genes also achieved the maximum AUC **(E)**, with an AUC value of 0.690 **(F)**.

To validate the predictive value of the four candidate genes associated with oxaliplatin resistance, we constructed multivariate logistic regression models using various combinations of *AXDND1*, *BAMBI*, *MAPK8IP2*, and *BMP7* and tested them on three independent external datasets. A series of models, ranging from single-gene predictors to multi-gene combinations were evaluated based on their ROC-AUC values (**Figures 4A, C, E**). Among the models tested, the combination of *AXDND1, BAMBI*, and *BMP7* in the GDSC dataset achieved a notably high ROC-AUC value of 0.742 (**Figure 4B**). In the GSE83129 dataset, the inclusion of *MAPK8IP2* alongside the previous three genes further improved the ROC-AUC value to 0.812 (**Figure 4D**). Similarly, in GSE28702, this four-gene panel reached an ROC-AUC value of 0.690 (**Figure 4F**). These results consistently demonstrate that this gene cluster holds strong predictive potential for oxaliplatin sensitivity in CRC cells and patient samples.

### Validation of gene expression in CRC cell lines

**Figure 5:**
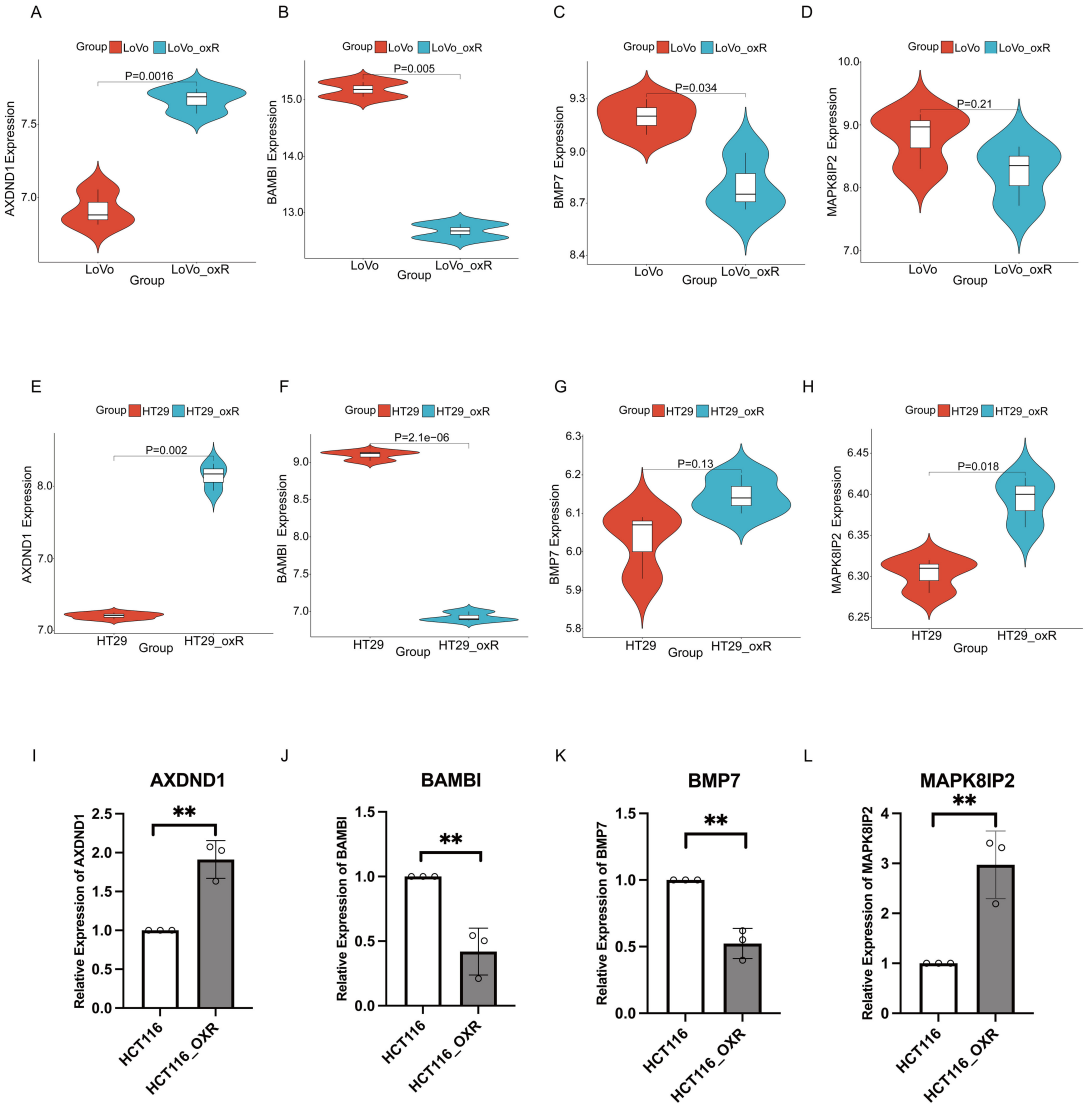
Validated the expression of key genes in oxaliplatin resistant CRC cells via external datasets. The validation of expression of 4 key genes in LOVO and LOVO oxaliplatin-resistant (LOVO_oxR) of GSE42387 dataset validation **(A–D)** or HT29 and HT29 oxaliplatin-resistant (HT29_oxR) of GSE76092 dataset **(E–H)**. **(I–L)** Detection of expression levels of 4 key genes in HCT116 and oxaliplatin-resistant CRC cell(HCT116_oxR) by qRT-PCR. The symbol ** indicates a statistical significance level of p<0.01.

To assess the robustness of the four core genes across different CRC models, we examined their expression profiles in oxaliplatin-resistant cell lines from external datasets GSE76092 (HT29_oxR) and GSE42387 (LOVO_oxR). In LOVO_oxR cells, *AXDND1* expression was significantly increased relative to parental controls (**Figure 5A**), consistent with its correlation with poor clinical outcomes. In contrast, both *BAMBI* (**Figure 5B**) and *BMP7* (**Figure 5C**) were markedly downregulated in the resistant cells, which aligns with their association with improved PFS. Meanwhile, *MAPK8IP2* expression did not differ significantly in this model (**Figure 5D**). In HT29_oxR cells, *AXDND1* (**Figure 5E**) and *MAPK8IP2* (**Figure 5H**) were significantly upregulated upon acquisition of resistance, while *BAMBI* (**Figure 5F**) was again significantly down regulated upon acquisition of resistance. As the gene selection process was initially based on differential expression in the HCT116_oxR line, we also performed qRT-PCR validation in this model. The expression patterns observed were consistent with prior predictions, reinforcing the relevance of these genes in oxaliplatin resistance (**Figures 5I–L**).

### Functional validation of AXDND1, BAMBI, BMP7 and MAPK8IP2

**Figure 6:**
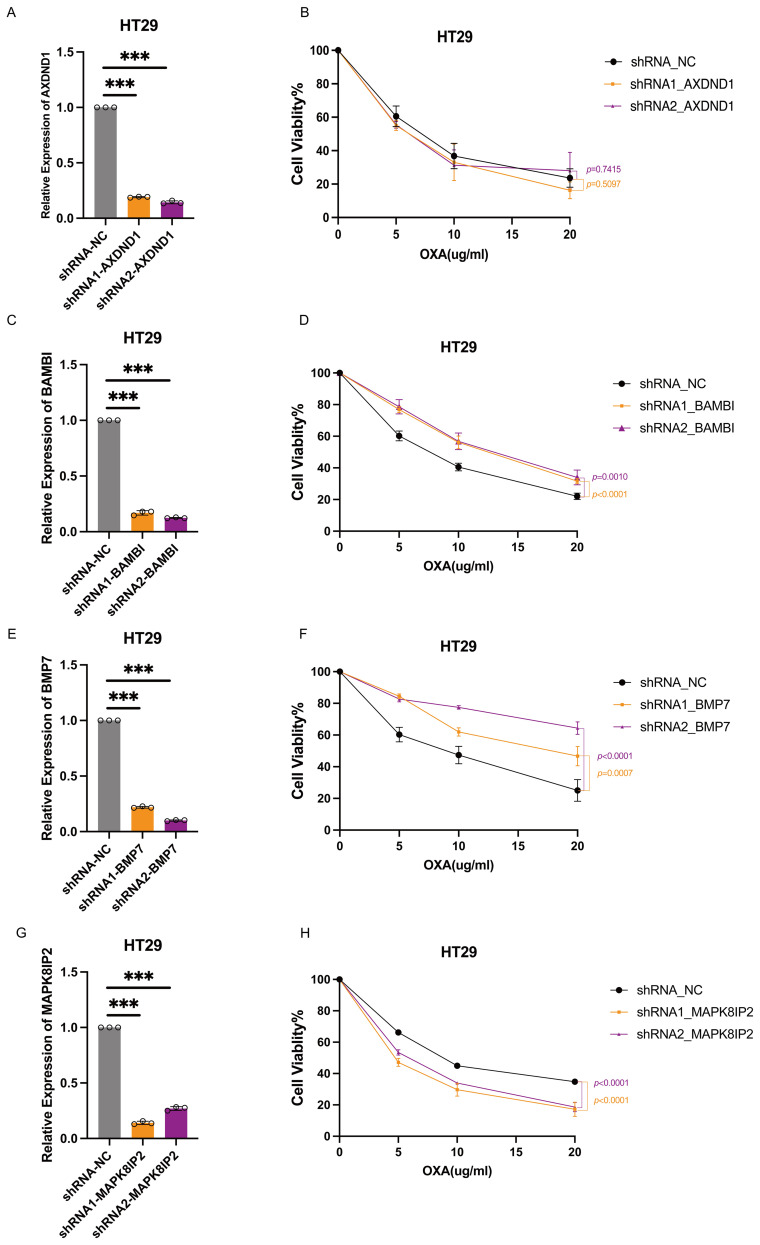
Impact of AXDND1, BAMBI, BMP7 and MAPK8IP2 knockdown on oxaliplatin sensitivity in HT29 cells. HT29 Cells were transduced by lentiviruses with shNC, shAXDND1, shBAMBI and shBMP7 infection, followed by qRT-PCR analysis of the indicated genes **(A, C, E, G)**. 8000 Cells were grown in a 96-well plate, and post-oxaliplatin treatment for 48 hours, cell viability was measured with the CCK8 **(B, D, F, H)**. Experiments in **(B, D, F, H)** were independently repeated three times for 3 repetitions with similar results, and the results of one representative experiment are shown, data represent means± s.d. Statistical significance was determined by two-way ANOVA. The symbol *** indicates a statistical significance level of p<0.001.

**Figure 7:**
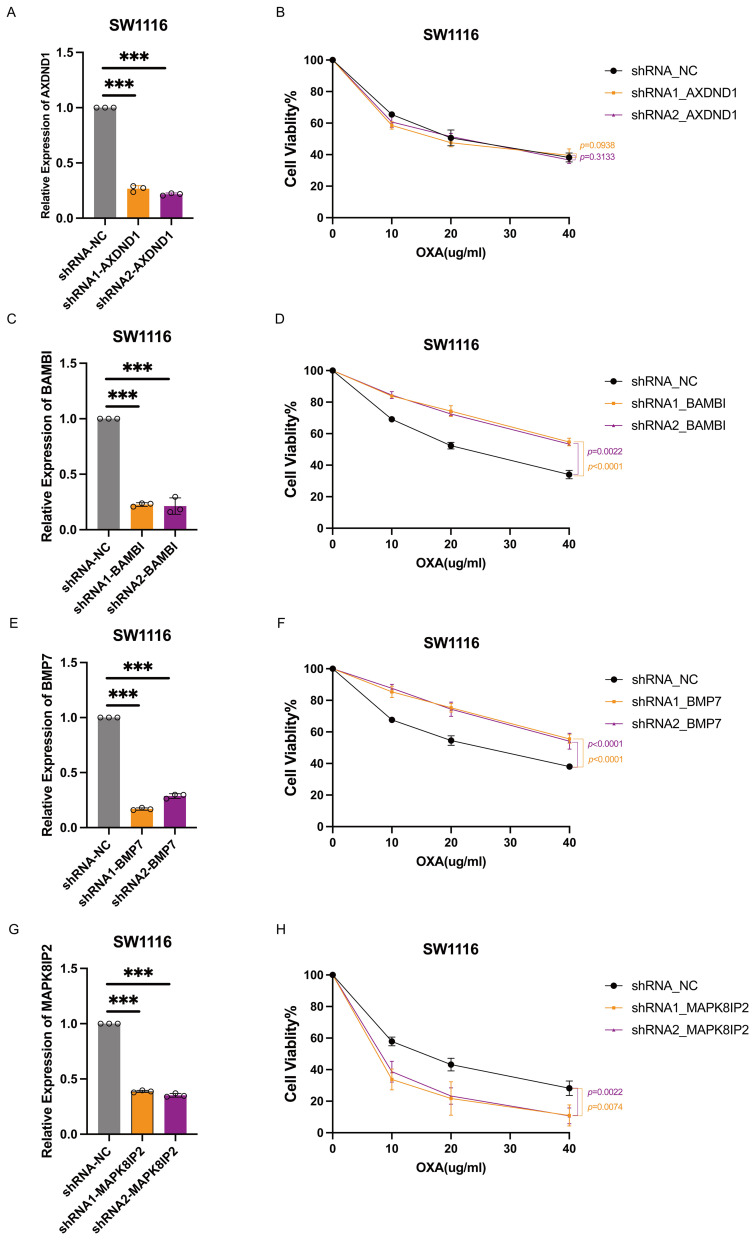
Impact of AXDND1, BAMBI, BMP7 and MAPK8IP2 knockdown on oxaliplatin sensitivity in SW1116 cells. SW1116 Cells were transduced by lentiviruses with shNC, shAXDND1, shBAMBI and shBMP7 infection, followed by qRT-PCR analysis of the indicated genes **(A, C, E, G)**. 4000 Cells were grown in a 96-well plate, and post-oxaliplatin treatment for 48 hours, cell viability was measured with the CCK8 **(B, D, F, H)**. Experiments in **(B, D, G, H)** were independently repeated three times for 3 repetitions with similar results, and the results of one representative experiment are shown, data represent means± s.d. Statistical significance was determined by two-way ANOVA.

In order to investigate the functional contribution of these genes to oxaliplatin sensitivity, we generated stable knockdown models in HT29 and SW1116 cells using shRNA technology. Efficient silencing was confirmed via quantitative reverse transcription PCR (qRT-PCR) (**Figures 6A, C, E, G**, **7A, C, E, G**). Following treatment with oxaliplatin, cell viability was assessed via CCK8 assays. Knockdown of *MAPK8IP2* significantly enhanced oxaliplatin sensitivity in both cell lines (**Figures 6H**, **7H**), whereas silencing *BAMBI* and *BMP7* led to increased resistance (**Figures 6D, F**, **7D, F**). In contrast, *AXDND1* knockdown produced no significant change in drug sensitivity (**Figure 6B**, **7B**), suggesting a less prominent or context-dependent role in the resistance mechanism.

## Discussion

By integrating transcriptomic data from the TCGA and GEO databases with machine learning algorithms (LASSO, SVM, and Random Forest), we identified a 14-gene panel predictive of oxaliplatin resistance in CRC. Among these, four genes (*AXDND1, BAMBI, BMP7*, and *MAPK8IP2*) showed significant associations with progression-free survival (PFS) in patients receiving oxaliplatin treatment. External dataset validation and functional experiments confirmed that silencing *BAMBI, MAPK8IP2*, or *BMP7* directly altered oxaliplatin sensitivity in CRC cell lines, supporting their potential as predictive biomarkers.

A key question emerging from our findings is whether this four-gene signature reflects a mechanism specific to CRC or a broader, pan-cancer mechanism underlying oxaliplatin response. To address this, we extended our analysis to other oxaliplatin-treated malignancies—specifically pancreatic adenocarcinoma (PAAD) and stomach adenocarcinoma (STAD)—using transcriptomic and clinical data from TCGA (**Supplementary Figures S3, S4**). In cohorts of 20 PAAD and 17 STAD patients, none of the four genes showed a statistically significant association with progression-free survival or differential expression between oxaliplatin-sensitive and resistant groups, though these results may be influenced by limited sample sizes. This context-dependent behavior aligns with the understanding that drug response mechanisms are often tissue-specific, shaped by distinct genetic backgrounds and tumor microenvironments (19). Thus, while the four-gene signature demonstrates robust predictive value in CRC, its lack of association in PAAD and STAD suggests that it is not a universal biomarker but may reflect a mechanism more specifically operative in colorectal cancer.

Although each of these genes has been individually associated with tumor biology, their combined role in oxaliplatin resistance remains poorly understood. BAMBI, a known antagonist of the TGF-β pathway, typically suppresses epithelial–mesenchymal transition (EMT) and tumor invasion. Previous studies indicated that *BAMBI* downregulation can enhance TGF-β signaling, promoting EMT in non-small cell lung cancer (20), and contributing to platinum resistance in epithelial ovarian cancer (21). Consistent with these findings, our data revealed that knocking down *BAMBI* decreased oxaliplatin sensitivity in CRC cells, likely via EMT activation.

MAPK8IP2, a scaffold protein in the JNK signaling cascade, has been linked to stress response and poor prognosis in prostate cancer (22). Our results showed that *MAPK8IP2* knockdown sensitized CRC cells to oxaliplatin, suggesting its involvement in cell survival and DNA repair mechanisms under chemotherapy-induced stress.

BMP7, a member of the TGF-β superfamily, primarily functions as a tumor suppressor. Reduced BMP7 expression has been correlated with tumor progression and drug resistance across multiple cancers. In CRC, BMP7 variants have been shown to restore chemosensitivity (23), while in breast cancer, higher BMP7 levels correlated positively with favorable immune profiles and prognosis (24). Our study aligns with these observations, demonstrating that knocking down *BMP7* increased oxaliplatin resistance in CRC models.

In contrast, AXDND1 remains largely unexplored in cancer biology. Initially identified in testicular development and spermiogenesis (25), its cancer-related functions remain unclear. Although we observed differential AXDND1 expression in oxaliplatin-resistant CRC samples and a correlation with patient prognosis, functional assays indicated limited effects on oxaliplatin sensitivity, suggesting its role as a passive biomarker.

Beyond genomic alterations, oxaliplatin resistance is also shaped by complex systemic interactions. Metabolic shifts—such as increased aerobic glycolysis and deregulated fatty acid oxidation—enhance tumor cell adaptability to chemotherapeutic stress, providing alternative bioenergetic routes to maintain proliferation under cytotoxic pressure (26). Concurrently, the gut microbiota has emerged as a significant modulator of treatment efficacy. Changes in microbial composition and metabolite production, such as short-chain fatty acids, can influence intestinal barrier integrity, drug metabolism, and immune priming (27, 28). Several studies have demonstrated that specific bacteria, such as Akkermansia muciniphila, can improve oxaliplatin sensitivity by modulating glycolysis and enhancing immune activation in the tumor microenvironment (27). Immune components also play a critical role: tumor-associated macrophages and regulatory T cells can secrete immunosuppressive cytokines that dampen the cytotoxic response to chemotherapy, thereby reducing oxaliplatin-induced apoptosis (11, 12, 29).

These insights highlight that oxaliplatin resistance arises not solely from tumor-intrinsic genetic or epigenetic alterations, but from a broader interaction between the tumor and its surrounding systemic environment. While our study did not directly interrogate the microbiome, metabolic flux, or immune cell infiltration, the identification of *BAMBI* and *MAPK8IP2*—genes known to modulate TGF-β and stress-immune pathways—suggests their potential interface with these resistance mechanisms. This aligns with recent findings linking immune-metabolic signaling to chemoresistance phenotypes (30). Future studies should therefore adopt integrated multi-omics strategies, including immunogenomics, metabolomics, and metagenomics, to fully elucidate how tumor-extrinsic factors converge with gene regulatory networks in mediating oxaliplatin resistance.

This study employed an integrative approach to identify a clinically relevant four-gene signature that may inform oxaliplatin-based therapy in CRC. Nevertheless, it should be noted that there are several limitations to this study. The relatively limited sample size of resistant CRC patients and cell lines has a detrimental effect on statistical robustness. The present study has focused exclusively on the effects of four gene knockdown on drug sensitivity in colorectal cancer cells, without delving into the molecular mechanisms through which three of these key genes – BAMBI, MAPK8IP2, and BMP7 – regulate oxaliplatin sensitivity. Furthermore, the present analysis concentrated exclusively on transcriptomic alterations, whilst eschewing an exploration of other resistance-related mechanisms, including autophagy, immune evasion, and metabolic reprogramming. This may overlook non-transcriptional contributors to chemoresistance. While our *in vitro* assays support gene functionality, further validation in clinical samples and animal models is essential for confirming translational applicability. Future studies should thus incorporate multi-omic, spatial, and single-cell analyses to more thoroughly capture tumor heterogeneity and host-tumor interactions. Another potential limitation of our study is that we did not investigate the role of sodium channel activity or extracellular sodium concentration in modulating oxaliplatin sensitivity in our cell models. Evidence suggests that voltage-gated sodium channels promote oxaliplatin uptake by modulating membrane potential (31), and sodium-dependent transport is critical for cellular drug accumulation (32, 33). Thus, ion dynamics within the tumor microenvironment may substantially affect oxaliplatin uptake and response—a regulatory dimension not addressed here. Future work should incorporate assessments of ion channel function to bridge our genetic findings with these pharmacokinetic mechanisms.

## Conclusion

This study identifies a four-gene signature—*AXDND1, BAMBI, MAPK8IP2*, and *BMP7*—with predictive value for oxaliplatin sensitivity in CRC. Through integrated analysis of transcriptomic data and functional validation, we demonstrate that *BAMBI, MAPK8IP2* and *BMP7* actively influence drug response. These genes represent promising biomarkers for stratifying patients and optimizing chemotherapy regimens. Our findings advance current understanding of chemoresistance and lay the groundwork for biomarker-guided personalization of CRC treatment.

## Data Availability

The original contributions presented in the study are included in the article/**Supplementary Material**. Further inquiries can be directed to the corresponding authors.
