# Supplementary material for: Identification of a signature gene set for oxaliplatin sensitivity prediction in colorectal cancer
Source: Front Oncol. 2025 Nov 27;15:1701328. doi: 10.3389/fonc.2025.1701328 (PMC12696748; doi:10.3389/fonc.2025.1701328)
Supplement: Supplementary file 1 [file DataSheet1.pdf]

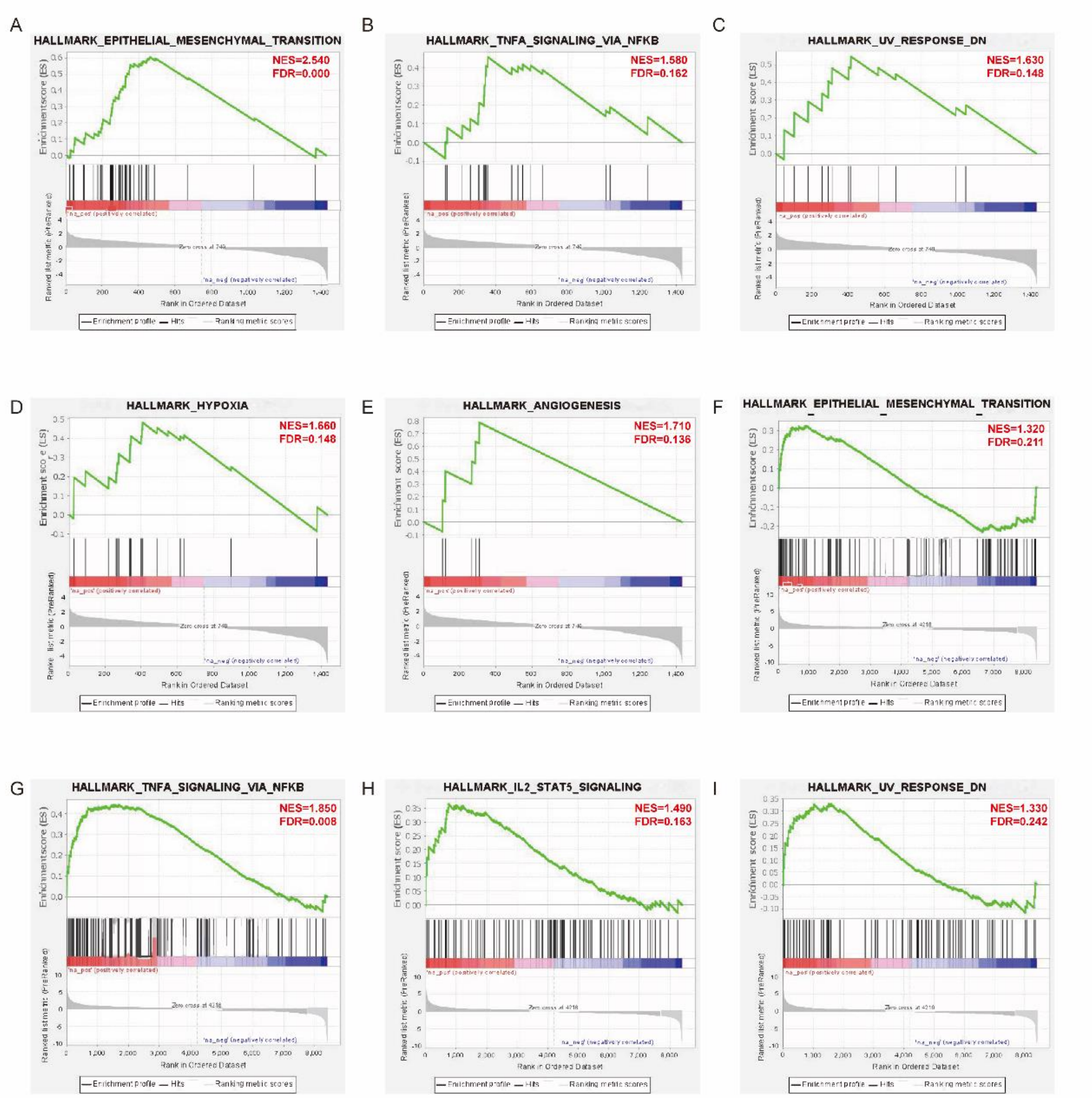

Supplementary Figure 1. Gene set enrichment analysis reveals key pathways associated with oxaliplatin resistance in CRC. GSEA was performed on ranked gene lists (Log2 fold-change) from differentially expressed genes (DEGs) in oxaliplatin-resistant versus -sensitive colorectal cancer samples derived from TCGA-COADREAD (A–E) and GSE119603 HCT116_oxR cells (F–I), using the MSigDB Hallmark gene set (h.all.v2025.1.Hs.symbols.gmt). Significant enrichment was defined as normalized enrichment score |NES| > 1 and FDR q-value < 0.25.
