# Supplementary material for: Identification of a signature gene set for oxaliplatin sensitivity prediction in colorectal cancer
Source: Front Oncol. 2025 Nov 27;15:1701328. doi: 10.3389/fonc.2025.1701328 (PMC12696748; doi:10.3389/fonc.2025.1701328)
Supplement: Supplementary file 2 [file DataSheet2.pdf]

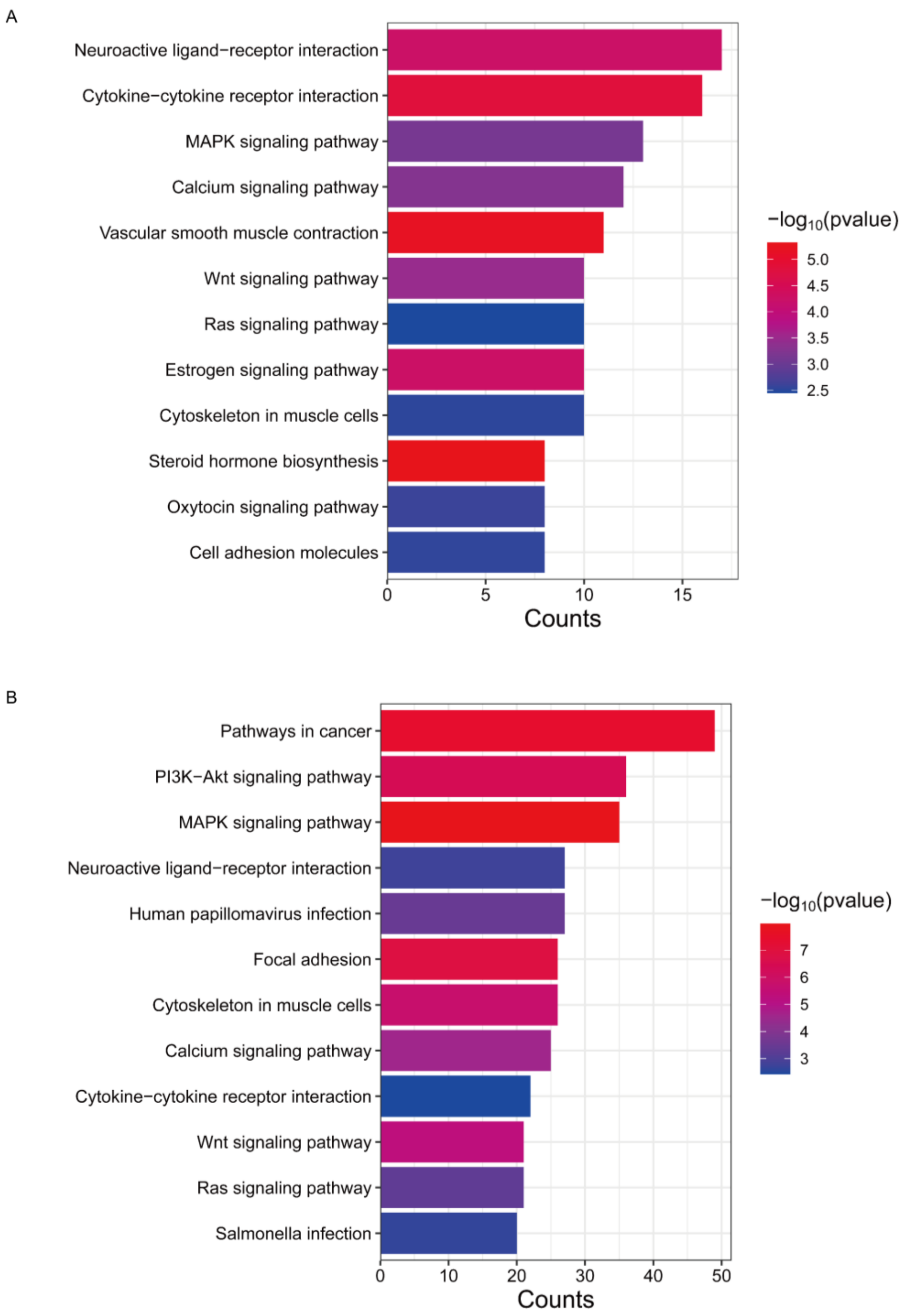

Supplementary Figure 2. KEGG pathway enrichment analysis of DEGs in oxaliplatin-resistant CRC. (A) Enriched KEGG pathways in DEGs identified between oxaliplatin-resistant and -sensitive colorectal cancer patients (n = 47; 19 resistant, 28 sensitive) from the TCGA-COADREAD cohort. (B) Enriched KEGG pathways in DEGs identified between oxaliplatin-resistant HCT116_oxR cells and parental controls (n = 3 per group) from the GSE119603 dataset. DEGs were determined using DESeq2

($|\log_2$ fold-change$| \geq 1$, $P \leq 0.05$); pathways with Benjamini-Hochberg adjusted $P < 0.05$ were considered significantly enriched.
