# Supplementary material for: Identification of a signature gene set for oxaliplatin sensitivity prediction in colorectal cancer
Source: Front Oncol. 2025 Nov 27;15:1701328. doi: 10.3389/fonc.2025.1701328 (PMC12696748; doi:10.3389/fonc.2025.1701328)
Supplement: Supplementary file 3 [file DataSheet3.pdf]

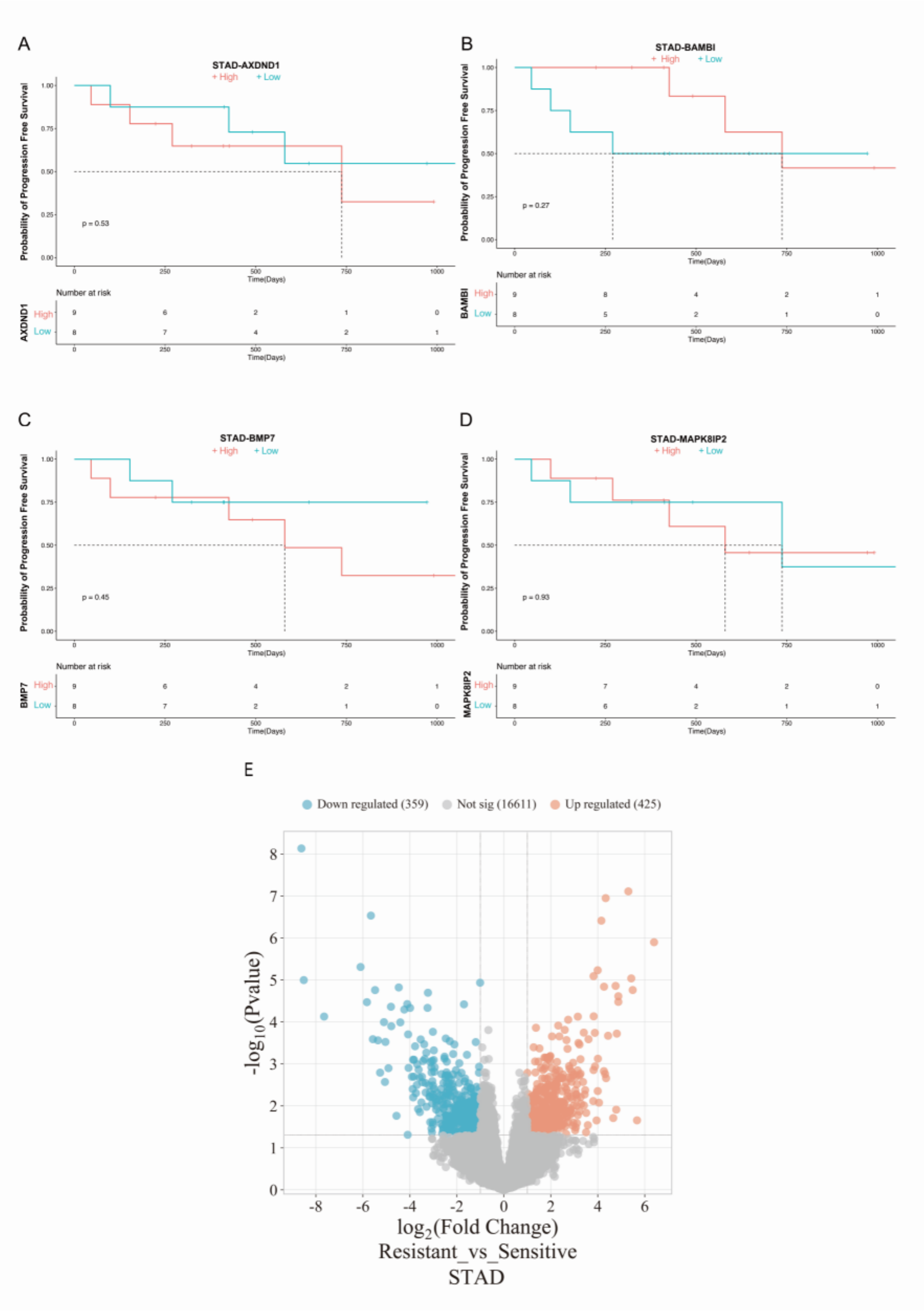


**Supplementary Figure 3. Evaluation of the four-gene signature in oxaliplatin-treated stomach adenocarcinoma (STAD).** (A) Volcano plot of differentially expressed genes (DEGs) between resistant and sensitive patients, using thresholds of |Log2 fold-change| ≥ 1 and P-value < 0.05. The four signature genes (AXDND1,

BAMBI, BMP7, MAPK8IP2) were not identified as significant DEGs. (B-E) Kaplan-Meier curves for progression-free survival (PFS) based on the expression of AXDND1 (B), BAMBI (C), BMP7 (D), and MAPK8IP2 (E). No significant associations were observed (log-rank test).
