# Supplementary material for: Identification of a signature gene set for oxaliplatin sensitivity prediction in colorectal cancer
Source: Front Oncol. 2025 Nov 27;15:1701328. doi: 10.3389/fonc.2025.1701328 (PMC12696748; doi:10.3389/fonc.2025.1701328)
Supplement: Supplementary file 4 [file DataSheet4.pdf]

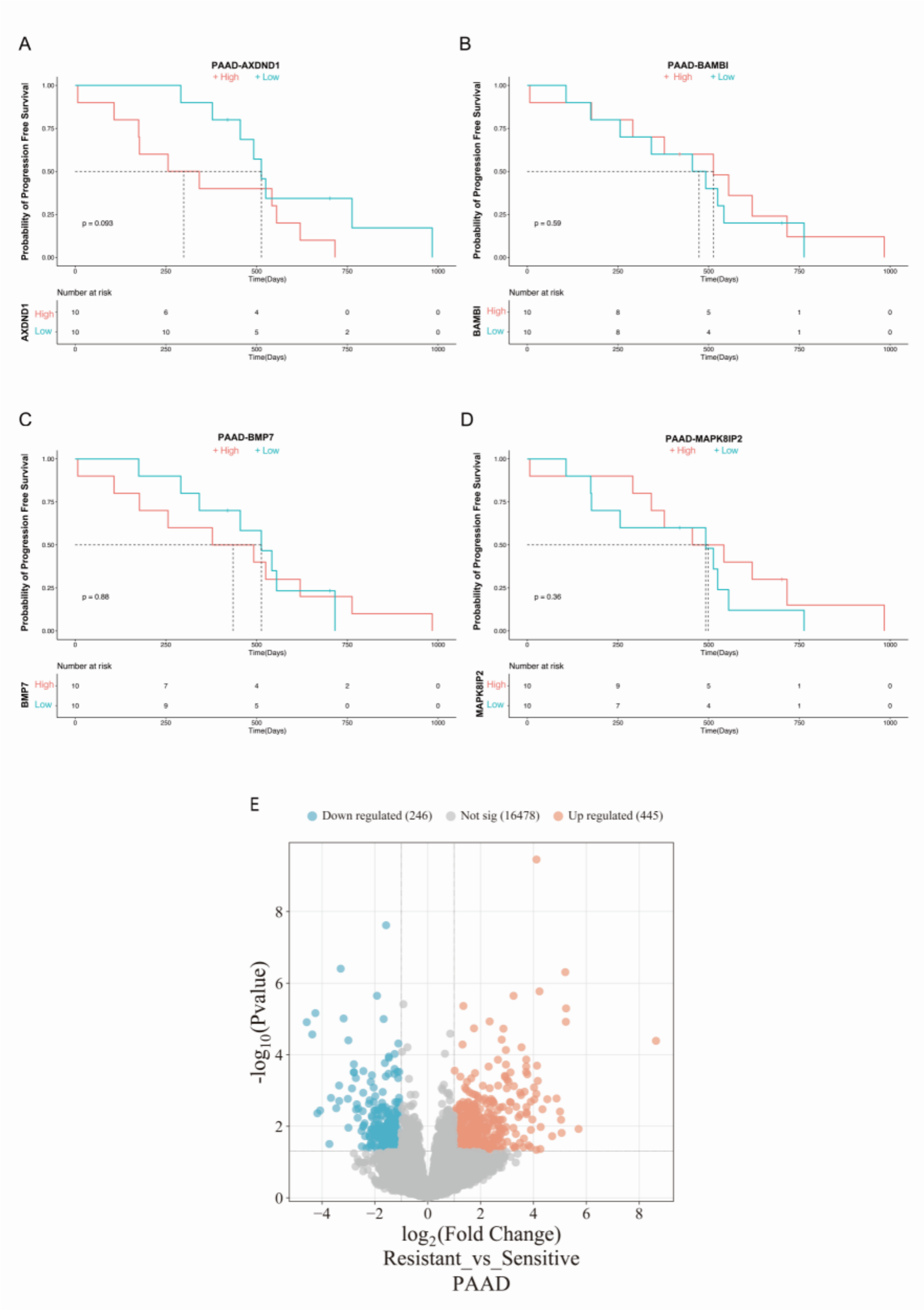

Supplementary Figure 4. Evaluation of the four-gene signature in oxaliplatin-treated pancreatic adenocarcinoma (PAAD). (A) Volcano plot of DEGs in the TCGA-PAAD cohort, with thresholds of |Log2 fold-change| ≥ 1 and P-value < 0.05.

None of the four signature genes showed significant differential expression. (B-E) Kaplan-Meier curves showing no association between the expression of AXDND1 (B), BAMBI (C), BMP7 (D), or MAPK8IP2 (E) and progression-free survival by the log-rank test.
